# Supplementary material for: Stochastically structured illumination microscopy scan less super resolution imaging
Source: Npj Imaging. 2024 Nov 6;2:45. doi: 10.1038/s44303-024-00047-x (PMC11541201; doi:10.1038/s44303-024-00047-x)
Supplement: Supplementary file 1 — Supplementary Information [file 44303_2024_47_MOESM1_ESM.pdf]

Stochastically Structured Illumination Microscopy scan less super resolution imaging; Supplementary Information

Denzel Fusco,[1,2] Emmanouil Xypakis,[3,2] Ylenia Gigante,[2,4] Lorenza Mautone,[2,4] Silvia Di Angelantonio,[2,5,4] Giorgia Ponsi,[2,6] Giancarlo Ruocco,[2,1] and Marco Leonetti*[3,2,4]
(e-mail: marco.leonetti@cnr.it Denzel Fusco and Emmanouil Xypakis equally contributed to results presented here.)
[1]*Department of Physics, University Sapienza, I-00185 Roma, Italy*
[2]*Center for Life Nano- & Neuro-Science, Italian Institute of Technology, Rome, Italy*
[3]*Institute of Nanotechnology of the National Research Council of Italy, CNR-NANOTEC, Rome Unit, Piazzale A. Moro 5, I-00185, Rome, Italy*
[4]*D-Tails s.r.l. BCorp, Via di Torre Rossa, 66, 00165 Rome, Italy.*
[5]*Department of Physiology and Pharmacology "V. Erspamer", Sapienza University of Rome, Rome, Italy.*
[6]*Department of Psychology, Sapienza University of Rome, 00185 Rome, Italy*
(Dated: August 23, 2024)

MEASUREMENT OF THE RETINAL SACCADIC MOVEMENTS DETAILS

Two volunteers (2 males, age = [43 25]), with normal or corrected-to-normal vision and without psychiatric or neurologic diagnoses, took part in the measurement of retinal saccadic movements. They were placed in a seated position in a dimly lit room and their head movements were restrained with a headrest. The eye tracker was positioned 55 cm from the participants. The task was displayed on a monitor (1920 × 1080 pixels, width: 38.2 cm, height: 21.5 cm, refresh rate: 120 Hz) placed behind the eye tracker and positioned 84 cm from the participants. Binocular eye movements were recorded using a high-resolution infrared eye tracker including a high-speed camera (35 mm lens), with a sampling rate of 1000/2000 Hz (EyeLink® 1000 Plus, SR Research Ltd., Ontario, Canada). The EyeLink 1000 Plus is a dark pupil-CR tracking system, i.e., it tracks both pupil center and CR (Corneal Reflection). This allows to have access to two points of reference on the eye to separate eye movements from head movements; in fact, the positional difference between pupil center and CR changes with eye rotation, but it remains relatively constant with minor head movements. The EyeLink 1000 Plus has a spatial accuracy of 0.25-0.50° (down to 0.15°) and a resolution (RMS, root mean squared) of 0.01° (0.05° for microsaccades, i.e. saccades smaller than 1 degree of visual angle. The eye tracker recorded horizontal and vertical gaze position in pixels on the screen (0,0 corresponds to the top-left of the screen). Before the task, calibration and validation procedures (provided by the eye tracker manufacturer) were achieved displaying 13 targets for the participants to fixate at these coordinates [(960,540), (960,92), (960,988), (115,540), (1805,540), (115,92), (1805,92), (115,988), (1805,988), (538,316), (1382,316), (538,764), (1382,764)]. During the calibration, the pupil-CR position for each target was recorded, and the set of target and pupil-CR positions were used to compute gaze positions during the recording. To validate the calibration, eye angles computed during validation were compared to the eye angles computed during the calibration phase. Participants had to repeat the procedures until the system determined the validation was good (maximum error for each point < 1°; average error < 0.5°), ensuring acceptable spatial accuracy. During the recording, pupil threshold and CR values were kept constant between 75-100 and below 230, respectively. The methodology followed the latest reporting guidelines in eye tracking research (Dunn et al., 2023, see ref. in the main paper). In the fixation task (see Supplementary Figure 1), each trial started with a black fixation cross (0.53° x 0.28°, 39 x 21 pixels) appearing at the centre of the screen (960,540), on a grey coloured background (R = 180, G = 180, B = 180). Participants had to look at the fixation cross and maintain their eyes on it for at least 300 ms. If they failed this gaze-contingent procedure within 4000 ms, the trial was discarded and recycled later during the experiment, and a new validation procedure was initialized. Afterwards, a black fixation dot (0.27° x 0.27°, 20 x 20 pixels) appeared at the centre of the screen (960,540) for a variable time interval ranging from 1000 to 15000 ms (15 trials with a relative time increase of 1000 ms each). The order of presentation of the trials was randomized. Participants were instructed to look at the fixation dot until its disappearance, by maintaining fixation on the target as much as possible. Finally, a blank screen was presented for 100 ms. Stimuli presentation was programmed and controlled through EyeLink® Experiment Builder (SR Research). Eye tracking data extraction was initially performed with EyeLink® Data Viewer.

RESULTS ON RETINA NEURON SAMPLE

In Supplementary Fig. 2, 3, 4 we report an additional field of view for retinal neurons samples Like in figure 4 of the main paper and in 5 we report an additional field for the test target.

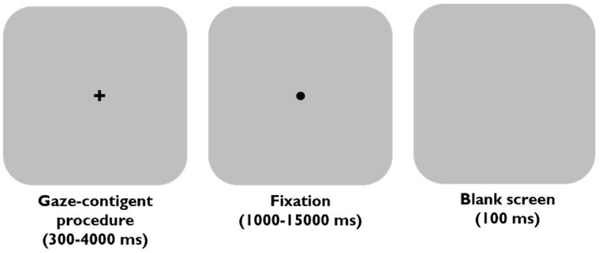

Supplementary Figure 1. Schematic representation and timeline of the fixation task.

IMAGE COMPARISON WITH ORDINARY MICROSCOPE

To evaluate the fidelity of the super-resolution reconstruction in the case of a biological signal (neuron culture) we compared an field imaged on the BIME (native resolution of 8 μm, Numerical Aperture of 0.035) with S^2IM, with the same image field measured with an higher resolution objective (Thorlabs RMS10X from Olimpus, Resolution of 1.3 μm Numerical Aperture of 0.25). Bare BIME imaging, S2IM and the 10X microscope images are reported in figure 6. The image profiles are along the segments reported in panel 6a are reported in 7. This plots show how feature absent in the wide field image are recovered with S^2IM. Moreover the same panels testify how the same features are present in the classical imaging data retrieved with an high numerical aperture objective. Indeed, the blue dashed curves represent the native smooth profiles of Low Resolution data from the BIME. A proxy for the Ground Truth is obtained with a 10X 0.25 NA objective, which show the presence of some features evidently "blurred away" by the poor quality of the ocular imaging. Thick red curves show how these features are retrieved thanks to the S^2IM super resolution.

Resolution measurement

To measure the resolution improvement of the super-resolution algorithm we employ the strategy of [1]. We use as a target ground truth object (GT) image a periodic signal

$$g(\theta) = 1 + cos(96\theta) = ||\hat{g}_0|| + ||\hat{g}_{96}||cos(96\theta), \quad (1)$$

where θ is the angle from the center, the $\hat{}$ symbol is used for the Fourier transform and $||||$ is the absolute value. To find the resolution improvement of the super-resolution algorithm we make use of the the disc of confusion of radious R defined as the minimum radious in which the fourier transform contrast of the

$$FTC = 2||\hat{f}(96)||/||\hat{f}(0)||, \quad (2)$$

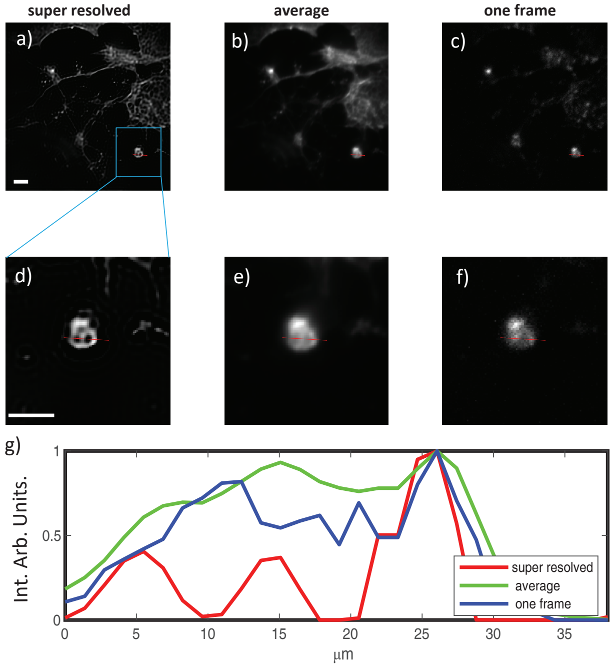

Supplementary Figure 2. Images relative to retinal neurons culture actine-stained and loaded on the m-BIME. Panels a-c) Report (from left to right), S^2IM, smart averaging, and single shot on the first field. Panels a-c) Report (from left to right), S^2IM, smart averaging, and single shot. Panels h-l) are organized as panels a-c). Panels d-f) represent zoom in of the areas highlighted by the blue square. Intensity profiles along the blue-red lines is reported in Panel g. Exposure time is 2ms, and $N = 2000$ single shot images have been acquired. Scale bars are all by 40 μm.

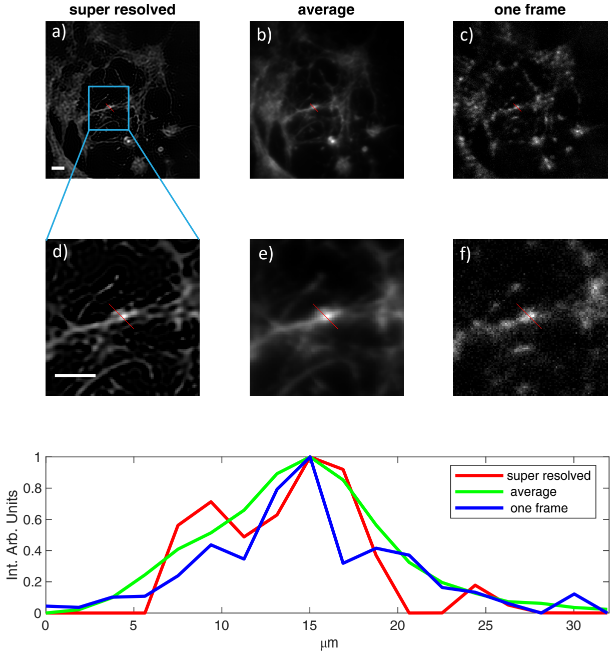

Supplementary Figure 3. Images relative to a retinal neurons culture (same properties as Supplementary Figure 2) but for different sample/area.

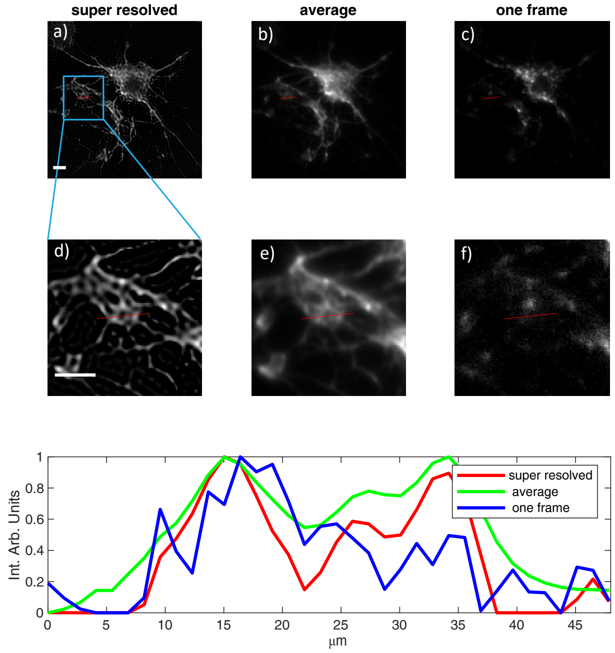

Supplementary Figure 4. Images relative to a retinal neurons culture (same properties as Supplementary Figure 2) but for different sample/area.

is above 0.1

With this criterion, we define the resolution improvement in the main text to be the ratio R_{LR}/R_{al}, where R_{al} is the radious value for different algorithms.

Robustness of S2IM to Noise

In order to model the signal noise in our system we take into account the read out noise and the photon noise. The noisy low resolution images are prepared by treating the readout noise as a Gaussian distributed noise $\mathcal{N}(\mu, \sigma_c^2)$ with the mean μ corresponding at the camera offset and $\sigma_c = 2$ camera counts. The photon noise is a Poissonian noise $\mathcal{P}(N_{ph})$ which mean corresponds to the total number of photons N_{ph} arriving at each camera pixel. Thus, the noisy LR image is

$$LR_{noisy} = \mathcal{P}(LR) + \mathcal{N}(100, 2) \tag{3}$$

The SNR parameter reported in figure 2 of the main paper, represents the signal to noise calculated as $SNR = \sqrt{N_{ph}} + \sigma_c$

In order to estimate how the performance of the algorithm is robust to an image registration error we add a guassian distributed position error to the image registration coordinates that is characterised by σ. The results are shown in figure 8.

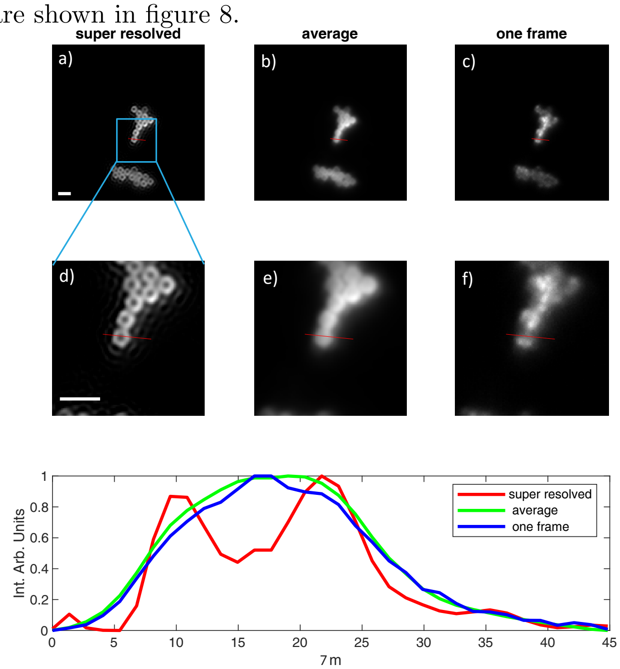

Supplementary Figure 5. Images relative to the test target object. Scale bar and experiment charateristics as described in Supplementary Figure 2 Caption.

[1] E. Xypakis, G. Gosti, T. Giordani, R. Santagati, G. Ruocco, and M. Leonetti, Scientific Reports **12**, 8623 (2022).

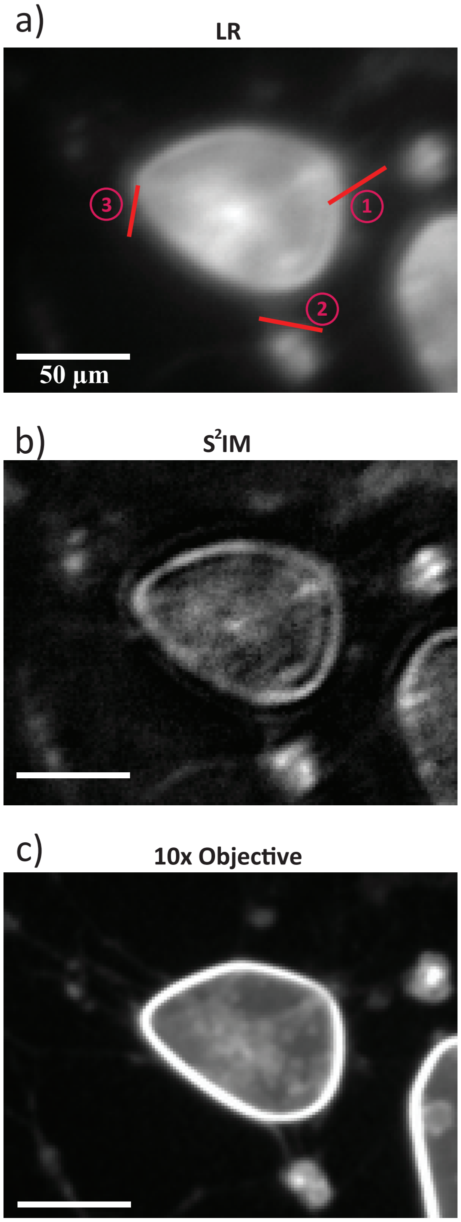

Supplementary Figure 6. Comparison between Wide field BIME image (panel a), S^2IM enhanced BIME Image (panel b) and 10× Objectives (panel c). Scale bars represent 50 μm. The sample consists of retinal Neurons. The red lines in panel a) represent the locations where the intensity profiles reported in figure 7 are measured.

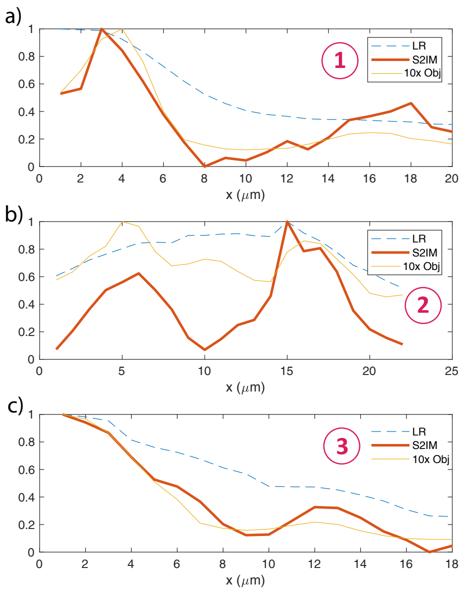

Supplementary Figure 7. Intensity profile relative to the segments highlighted in 6a. Dashed blue line is relative to the low resolution, smart averaged images from BIME. Thick orange line is obtained with the S^2IM super resolution of the BIME data and yellow line is relative to the 10X Objective (a good proxy of the real distribution of the fluorescence).

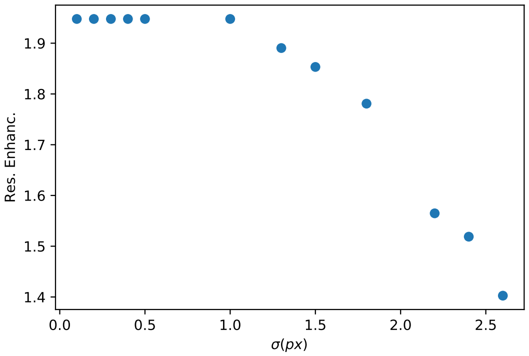

Supplementary Figure 8. The resolution enhancement Res. Enhance. versus the image registration standard deviation error σ measured in pixels
